# Infant and young child feeding practice among mothers with 0–24 months old children in Slum areas of Bahir Dar City, Ethiopia

**DOI:** 10.1186/s13006-017-0117-x

**Published:** 2017-06-14

**Authors:** Yeshalem Mulugeta Demilew, Tadese Ejigu Tafere, Dereje Berhanu Abitew

**Affiliations:** School of Public Health, College of Medicine and health Sciences, Bahir Dar University, P.O. Box 79, Bahir Dar, Ethiopia

**Keywords:** Complementary feeding, Infant and young child, Breastfeeding

## Abstract

**Background:**

Adequate nutrition during infancy and early childhood is essential to ensure the health, growth and development of children. However, infant feeding practice is suboptimal in Bahir Dar City, Ethiopia. The slum area is a heavily populated urban informal settlement characterized by substandard housing, squalor, with a lack of reliable sanitation services, supply of clean water, reliable electricity, law enforcement and other basic services. Residents of the slum area were poor and less educated. This further compromises infant feeding practice. The aim of this study was to assess infant and young child feeding practice among mothers with 0–24 month old children in the study area.

**Methods:**

A community based cross-sectional study was conducted among 423 mothers with 0–24 month old children from June 01-30 / 2016. Simple random sampling technique was used to select the respondents. Infant and young child feeding practice was assessed using the fifteen World Health Organisation (WHO) criteria.

**Results:**

The prevalence of exclusive breastfeeding practice was 113 (84%). Sixty (15%) mothers gave prelacteal feeds and, 96 (23%) mothers used a bottle to feed their index child. Appropriate complementary feeding practice was only 20 (7%). Thirty nine out of forty mothers introduced complementary food timely, 131 (47%) of mothers gave the minimum meal frequency, and 20 (7%) children took the minimum food diversity and acceptable diet. Independent predictors for complementary feeding practice were having secondary and above education of the mother, receiving postnatal care, possession of radio and giving birth at hospital.

**Conclusion:**

In this study infant and young child feeding (IYCF) practice was poor. Therefore, there is a need for strengthening the promotion on IYCF practice during postnatal care and using mass media to giving﻿ emphasis for ﻿optimal complementary feeding practices, especially for mothers with a lower educational status.

## Background

Maternal and child undernutrition remain pervasive and damaging in low and middle-income countries [1, 2]. Globally, more than a third of child deaths and above 10 % of the disease burden are attributable to maternal and child undernutrition [3]. Additionally, early undernutrition has long lasting effects on physical as well as cognitive growth of the child [3, 4].

Adequate nutrition during infancy and early childhood is essential to ensure the growth, health and development of children to their full potential [5]. Hence, the first two years of life provide a critical window of opportunity for prevention of growth faltering and undernutrition through optimal feeding [6, 7]. Improving infant feeding practices especially for children younger than two years of age should therefore be a high global priority [6].

World Health Organization (WHO) and United Nations Children’s Fund (UNICEF) set a global strategy for optimal infant and young child feeding (IYCF) [8]. The strategy recommends the initiation of breastfeeding within one hour of birth, exclusively breastfed for the first six months, after which nutritiously appropriate, adequate, and safe complementary foods should be introduced along with continuing breastfeeding up to two years and beyond. Improving IYCF practices based on this recommendation when children are well and sick is important to ameliorate undernutrition and its consequences [5, 9].

Infant and young child feeding practice is suboptimal throughout the world [10], especially the late initiation of breastfeeding, prelacteal feeding, early or late introduction of optimal complementary foods, giving poor quality, quantity and unhygienic complementary food, and using a bottle to fed the child are the common practices in developing countries [10–14].

The Ethiopian government also developed and implemented the IYCF guideline in 2004 to improve feeding practice [15]. However, the IYCF practice remains inappropriate and likely to be a major cause of under nutrition [16]. According to the 2016 Ethiopian Demographic and Health Survey (EDHS), infant and young child feeding practices are not as recommended by WHO. Only 58% of infants less than six months of age are exclusively breastfed and the optimal complementary feeding practice was 7%. Contrary to the recommendation by WHO. Nine percent of infants less than six months of age use a bottle with a teat, a practice that is discouraged because of the risk of illness to the child [17].

Furthermore, the optimal feeding practice is low in Amhara region where 38% of neonates start breastfeeding within one hour of birth and one in three (34%) children are fed the minimum meal frequency per day [2]. Only, 2.1% of children received the minimum dietary diversity and minimum acceptable diet. Feeding practice is poor especially in slum areas as they are densely populated informal settlements with substandard housing conditions, poor environmental hygiene and more likely to be uneducated or less educated people [18]. Therefore, this study was conducted to assess current status of IYCF practice and associated factors in slum areas of Bahir Dar City.

## Methods

The study was conducted in slum areas of Bahir Dar City from June 1–30, 2016. The City is the capital City of Amhara Regional State, which is found at 565 km far from Addis Ababa, Northwest Ethiopia. The total population in the City is 288,200, of these, 146,982 are females. For administrative purpose the City is divided in to nine sub-cities. Among which, three sub-cities (Shumabo, Gish-Abay and Sefene-selam) are slums. The majority of the residents in the slum areas are daily laborers and petty traders. According to the Bahir Dar City administration health office Bureau, the number of children under-five years of age and children from 0 to 24 months old were 4389 and 1665 respectively [19].

A community based cross-sectional study was conducted among mothers who had children aged between 0 and 24 months. The sample size was determined using the single population proportion formula by considering an assumption of: 95% confidence level, and the proportion of exclusive breastfeeding 52% from a previous study [2], marginal error of 5% and 10% nonresponse rate. The final sample size was 423. The final sample size was determined by considering exclusive breastfeeding since it gives a largest sample size compared to the other IYCF practices.

The sample frame was list of children (0–24 months of age) in the slum areas registered by the urban health extension workers. Using this registration logbook the study participants were selected by Simple random sampling technique (lottery method) considering proportional to size allocation (by considering the number of infant and young children) for each slum area. In households with two children less than two years of age, one was selected by lottery method.

Data were collected by an adapted, pretested, structured interviewer administered questionnaire. It was adapted from different literature and guidelines [5, 8–10]. The questionnaire was developed in English and translated to Amharic, back-translated to English by an independent translator for consistency. An interview with mothers of the index child was conducted at their home ensuring privacy. Three female diploma nurses and one public health professional were recruited as data collector and supervisor respectively.

Infant and young child feeding practice was assessed using eight core and seven optional feeding practice indicators developed by WHO to assess the adequacy of IYCF practices. The World Health Organisation defines optimal IYCF practice as the initiation of breastfeeding within one hour of birth, breastfeeding exclusively for the first six months, continuing to breastfeed for two years, on demand breastfeeding, giving of colostrum, no prelacteal feeding, no bottle feeding and initiation of solid and semi-solid food at six month, minimum dietary diversity, minimum meal frequency, minimum acceptable diet, consumption of iron-rich or iron-fortified foods, age-appropriate breastfeeding, predominant breastfeeding under six months, and milk feeding frequency for non-breastfed children [8–10]. All these indicators were assessed based on a 24-h recall method. In this study optimal feeding practice was assessed based on compliance to WHO recommended practices for each indicator. Complementary feeding practice was assessed based on compliance to WHO recommended practices for timely initiation (introduce complementary feed at six months), minimum meal frequency (fed minimum of three meals/day and four times/day for children aged 6–8 months and 9 months and above respectively) and minimum meal diversity (fed four or more foods within 24 h). Complementary feeding practice was considered appropriate if all the three indicators mentioned above were fulfilled otherwise it was considered as in appropriate.

Two days intensive training was given to the data collectors and the supervisor on techniques of data collection, instruments and how to maintain ethical issue. The pretest was done in similar settings but not included in the main study of 5% of the sample size. To assure the quality of the data, the supervisor and investigators closely reviewed the data collection technique on daily basis, reviewed the filled questionnaire for completeness and returned any incomplete questionnaire to the data collectors for correction. There was also debriefing every day.

Data were entered and analyzed using SPSS version 20. Descriptive statistics like frequency, proportions, mean and standard deviation were computed when necessary. In addition, bivariate and multivariable logistic regression was also carried out to see associations. Crude and Adjusted Odds ratios (COR, AOR) were computed for each explanatory variable to determine the strength of association and to control the confounders. The *p* value ≤0.2 was taken as a cut-off point to select eligible variables for the multiple logistic regression analysis and *p*-values <0.05 was considered statistical significant in the final model.

The study was approved by Institutional Review Board of Bahir Dar University. A letter of permission was given from Bahir Dar city administration health office and sub-city administrators. Verbal consent was taken from participants. Privacy and confidentiality was maintained throughout the study period by excluding personal identifiers during data collection.

## Results

### Sociodemographic characteristics

Among the 423 mothers, 412 participated in this study (97% response rate). The mean age of children and mothers was 11.39 (± 6.8 Standard Deviation [SD]) months and 27.68 (± 4.8 SD) years respectively. All respondents were Orthodox Christian followers by religion. The majority, 397 (96%) mothers were married and, 394 (96%) were from Amhara ethnic group. Above half, 226 (55%) children lived in male headed households (Table 1).

**Table 1:** Sociodemographic characteristics of study participants to assess IYCF practice in Slum areas of Bahir Dar City, Ethiopia, June 2016

Variable	Frequency (*n* = 412)	Percentage
Age of the mother (years)		
≤ 24	112	27
25–29	163	40
± 30	137	33
Place of birth		
Health center	241	58
Hospital	171	42
Ethnicity		
Amhara	394	96
Agew and Tigray	18	4
Educational status of the mother		
Have no formal education	121	29
Primary education	136	33
Above primary education	155	38
Occupational status of the mother		
Housewife	263	64
Petty trader and daily laborer	104	25
Government employee	45	11
Marital status of the mother		
Married	397	96
Never married	15	4
Occupational status of the father (*n* = 397)		
Daily laborer and Bajaji driver	194	49
Government employee	91	23
Carpenter and petty trader	112	28
Educational status of the father (*n* = 397)		
Have no formal education	66	17
Primary education	82	20
Above primary education	249	63
Family member		
≤ 3	184	45
> 3	228	55
Head of the household		
Both parents	186	45
The father only	226	55

One hundred twenty one (29%) mothers and fathers, 66 (17) have no formal education. Two hundred sixty three (64%) mothers were housewives and 194 (49) fathers were daily laborers and Bajaji drivers (Table 1). The majority, 397 (96%) of children lived with their both biological parents. The caregivers for 281 (68%) children were their mothers (Table 2).

**Table 2:** Sociodemographic characteristics of indexed children for this study to assess IYCF practice in Slum areas of Bahir Dar City, Ethiopia, June 2016

Variable	Frequency (*n* = 412)	Percentage
Sex of the child		
Male	222	54
Female	190	46
Age of the child (months)		
0–6	134	33
> 6–24	278	67
The child lives with		
Both biological parents	397	96
The mother only	11	3
Grandmother	4	1
Caregiver to the child		
The mother only	281	68
Mother and servant	64	16
Both biological parents	53	13
Grandmother	14	3

### Infant and young child feeding practice

**Fig. 1:**
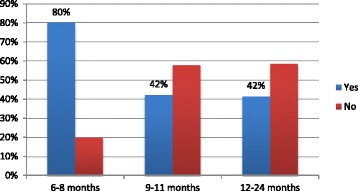
Minimum meal frequency by age of children in Slum areas of Bahir Dar City, Ethiopia, June 2016

**Fig. 2:**
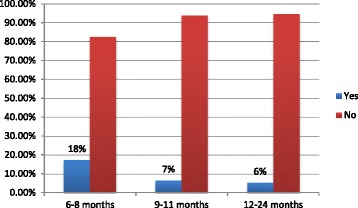
Minimum meal diversity by age of children in Slum areas of Bahir Dar City, Ethiopia, June 2016

From the total of 412 mother child pairs who participated in the study, 113 (84%) infants aged less than six months were exclusively breastfed. Among 65 mothers who had 12–15 months old children, 60 (92%) of them continued to breastfed their children at one year. Eighty eight children were aged from 20 to 24 months, and 83 (94%) of them continued to breastfed at age two years (Table 3). Sixty (15%) mothers gave prelacteal feeds and, 96 (23%) mothers used a bottle to feed their index child (Table 4). Among 40 mothers with children aged between 6 and 9 months old, 39 (97%) started giving complementary foods or drinks other than breast milk to their infants. Overall, 131(47%) children received the minimum meal frequency (Fig. 1 for specific age groups). In addition 20 (7%) of children aged between 6 and 23 months old have received the minimum meal diversity and minimum acceptable diet. The prevalence of appropriate complementary feeding practices was 20 (7%) (Fig. 2 for specific age groups).

**Table 3:** WHO criteria to assess infant and young child feeding practice in Slum areas of Bahir Dar City, Ethiopia, June 2016

Variable	Frequency (*n* = 412)	Percentage
Ever breastfed (0–23 months)		
Yes	397	96
No	15	4
Started breastfeeding within 1 h (0–23 months) (*n* = 397)		
Yes	338	85
No	59	15
Exclusively breastfed (0–6 months) (*n* = 134)		
Yes	113	84
No	21	16
Continued breastfeeding at one year (12–15 months) (*n* = 65)		
Yes	60	92
No	5	8
Started solid, semi-solid or soft foods at 6 month (6–23 months) (*n* = 40)		
Yes	39	97
No	1	3
Took minimum dietary diversity (6–23 months) (*n* = 278)		
Yes	20	7
No	258	93
Took minimum meal frequency (6–23 months) (*n* = 278)		
Yes	131	47
No	147	53
Took minimum acceptable diet (6–23 months) (*n* = 278)		
Yes	20	7
No	258	93
Consumed iron-rich foods (6–23 months) (*n* = 278)		
Yes	22	8
No	256	92
Continued breastfeeding at two years (20–23) (*n* = 88)		
Yes	83	94
No	5	6
Got age-appropriate breastfeeding (0-23 months)		
Yes	362	88
No	50	12
Predominantly breastfed (0–6 months) (*n* = 134)		
Yes	127	95
No	7	5
No bottle feeding (0-23 months)		
Yes	316	77
No	96	23
Non-breastfed children took at least two milk feeding (*n* = 15)		
Yes	12	80
No	3	20

**Table 4:** Additional criteria to assess infant and young child feeding practice in Slum areas of Bahir Dar City, Ethiopia, June 2016

Variable	Frequency (*n* = 412)	Percentage (%)
Received colostrum (0–23 months)		
Yes	347	84
No	65	16
Prelacteal fed (0–4 months)		
Yes	60	15
No	352	85
On breastfeeding during the time of data collection (0–23 months)		
Yes	386	94
No	26	6
On breastfed in the last24hours (0–23 months)		
Yes	381	93
No	31	7
Frequency of breastfeeding/24 h (0–23 months) (*n* = 381)		
≥ 8 times (on demand)	317	83
< 8times	64	17
Time of initiation of complementary food (6–23 months) (278)		
Before 6 month	23	8
At 6 month	217	78
After 6 month	38	14

**Fig. 3:**
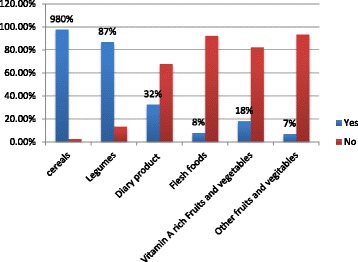
Type of food the children took in the last 24 h in Slum areas of Bahir Dar City, Ethiopia, June 2016

The majority, 271 (98%) and 241 (87%) children received cereal and legume based foods respectively. Ninety (32%) children received dairy products and 22 (8%) mothers gave flesh foods (meat) and 22 (8%) mothers gave iron rich foods for their children (Fig. 3).

### Factors associated with complementary feeding practice

The bivariate logistic regression analysis showed that place of delivery (hospital vs health center), age of the mother, maternal education, attending postnatal care, possession of radio and maternal occupation were statistically associated with complementary feeding practice (Table 5). In the multivariable logistic regression analysis, place of delivery, educational status of the mother, having postnatal care and possession of radio were independent predictors for complementary feeding practice. Mothers who have postnatal care follow up were four times more likely to have appropriate complementary feeding practice than those who did not attend postnatal care (Adjusted Odds Ratio[AOR] 4.1; 95% Confidence Interval [CI] 1.1, 7.3). Mothers who attend above primary education were three times more likely to have appropriate complementary feeding practice than their counterparts (AOR 3.0; 95% CI 1.2, 8.6). Mothers who have a radio were 3.2 times more likely to have appropriate complementary feeding practice than mothers who have no radio (AOR 3.2; 95% CI 1.1, 8.8). Mothers who delivered at hospital were 2.4 times more likely to have appropriate complementary feeding practice than mothers who gave birth at health centers (AOR 2.4; 95% CI 1.1, 7.3) (Table 5).

**Table 5:** Bivariate and multivariable logistic regression analysis of factors affecting complementary feeding practice of 6–24 months old children in Slum areas of, Bahir Dar City, Ethiopia, June 2016 (*n* = 278)

Variable	Complementary feeding practice		COR (95% CI)	AOR (95% CI)
	Appropriate (%)	Inappropriate *n* (%)		
Place of delivery				
Hospital	14 (5)	92 (33)	4.2 (1.5, 11.3)	2.4 (1.1,7.3)
Health center	6 (2)	166 (60)	1.00	1.00
Age of the mother (years)				
< 30	15 (5)	151 (54)	2.1 (0.7, 6.0)	
≥ 30	5 (2)	107 (39)	1.00	
Maternal education				
Less than secondary	7 (2)	186 (67)	1.00	1.00
Secondary and above	13 (5)	72 (26)	4.7 (1.8, 12.5)	3.0 (1.2, 8.6)
Family members				
≤ 3	6 (2)	119 (43)	1.9 (0.7, 5.3)	
> 3	14 (5)	139 (50)	1.00	
Attended PNC				
Yes	12 (4)	85 (31)	3.0 (1.2,7.7)	4.1 (1.4, 12.2)
No	8 (3)	173 (62)	1.00	
Have radio				
Yes	11 (4)	74 (27)	3.0 (1.1, 7.6)	3.2 (1.1, 8.8)
No	9 (3)	184 (66)	1.00	1.00
Maternal occupation				
Housewife	7 (2)	150 (54)	1.00	
Daily laborer/petty trader	8 (3)	85 (31)	2.0 (1.0, 5.7)	
Government employee	5 (2)	23 (8)	4.6 (1.4, 15.9)	

*COR* Crude odds ratio, *AOR* Adjusted odds ratio, *CI* Confidence interval

## Discussion

In this community based cross-sectional study 85% of mothers initiated breastfeeding within the first hour after delivery. This finding is higher than previous study findings in Ethiopia [20], Tanzania [21] and different parts of India [22, 23]. The discrepancy might be due to the time between studies and in Ethiopia, the number of mothers who give birth at a health institution is dramatically increasing due to persistent promotion of the free delivery service provision in the country, which creates a good opportunity for health professionals to promote the initiation of breastfeeding within an hour after birth.

About 84% of mothers who have children aged less than six months exclusively breastfed their index infant in the last 24 h. This practice is higher than previous study findings in Ethiopia [24–26], Tanzania [21, 27], Zambia [28] and India [22]. The difference might be due to socioeconomic and cultural difference between the study subjects. The majority of the participants in this study were housewives which could increase the likelihood of breastfeeding their child, as it cost less when they have an poor economic status.

Sixty (92%) and 83 (94%) mothers have continued to breastfeed their children at age one and two years respectively. This finding is consistent with previous study finding in Ethiopia [20]. However, it is higher than the study finding in Pakistan [29]. This difference might be due to the sociocultural difference between the study participants as breastfeeding for a long duration is traditional practice of Ethiopian mothers and currently there is increased promotion of breastfeeding. In addition the majority of women participated in the study were housewives and spend much of their time at home which increases the likelihood of continuing to breastfeed.

About 23% of mothers used a bottle to feed their index child which is not a WHO recommendation. A similar finding is reported from a previous study finding in Ethiopia [24] and studies in India [30, 31]. This might be because the majority of mothers had no formal education and a lack of access and exposure to mass media because of their poor socioeconomic status (from slum areas).

The prevalence of age appropriate breastfeeding is 88%. This finding is higher than the study finding in Pakistan [29] and might be due to a difference in study setting and time gap between studies.

Thirty nine (98%) mothers gave complementary foods or drinks other than breast milk to their infants. This finding is consistent with study finding in urban informal settlements in Nairobi, Kenya [32]. However, this finding is higher than the previous study findings in Ethiopia [33, 34], Pakistan [35], Mauritius [36] and India [30]. The difference might be due to the governmental and nongovernmental organizations who are currently promoting the benefit of complementary feeding through professionals and mass media.

About 47% of 6–23 months old children have been given the minimum meal frequency. This finding is consistent with the study findings in India [31] and Pakistan [37]. However, this finding is higher than the study finding in Nepal [38].

Overall, 20 (7%) of children aged between 6 and 23 months old have received the minimum meal diversity and minimum acceptable diet. This finding is consistent with study findings in Mumbai [39] and Pakistan [37]. Yet, this finding is lower than the study findings in other parts of Ethiopia [33, 40], Ghana [41] and India [31, 42]. This could be due to difference in study settings as this study was conducted in slum areas hence the participants were poor and with low educational status.

When the age of children increased, the proportion of children who received the minimum meal frequency, meal diversity and acceptable diet, decreased which is against the WHO recommendation. This necessitates the need to impart information on the quality of complementary food required by the child with respect to the age of the child.

The prevalence of appropriate complementary feeding practices is only 7%. This finding is consistent with study findings in Ethiopia [33] and Pakistan [37]. However, this finding is lower than the study finding in Nepal [38]. This discrepancy might be due to the socioeconomic differences like income and educational status between study participants.

About 98% and 87% of children aged between 6 and 24 months received cereal and legume based foods respectively. Moreover, a minority, 18% and 7% of children aged 6–24 months have received vitamin A rich and other fruits and vegetables respectively. This finding is consistent with a previous study finding in Ethiopia [20]. This might be because the participants of this study were low in economic status, and they preferred giving cereal based foods which are less expensive than animal products, fruits and vegetables.

Consumption of diary product, flesh foods like meat and iron rich foods is low. Only, 32% children took dairy products and 8% mothers gave flesh foods. Surprisingly, no one took egg in the last 24 h prior to the study. This finding is lower than study finding in Ethiopia [20] and Mumbai [39]. This difference might be due to difference in the study settings. Moreover, the price of animal products might be unaffordable for the poor population in slum areas.

A significant association was observed between appropriate complementary feeding practice and attending postnatal care service. This finding is consistent with previous study findings in Ethiopia [43], Kenya [44], Tanzania [45] and India [46]. This might be due to that reason that women who had post natal care visit might have highly likely to get education on IYCF practice during their visit.

The educational status of the mother had an association with appropriate complementary feeding practice. This finding is similar with previous study findings in Ethiopia [43], Pakistan [37] and Nepal [38]. This might be due to the fact that educated mothers have a better understanding of nutrition education than less educated mothers or mothers without formal education. Additionally, educated mothers might read books, leaflets and magazines, and might have a better chance of exposure to nutrition education about IYCF through mass media than their counter parts.

Possession of radio was an independent predictor for complementary feeding practices. This finding is in agreement with study finding in Tanzania [45]; mothers who have radio are more likely to be exposed to IYCF education provided through mass media.

The type of institution where the mothers give birth was another predictor for complementary feeding practices. This finding is consistent with the study finding in India [30]. Mothers who gave birth at the hospital are managed by professionals with better qualification than mothers who delivered in the health centers. Additionally, in the hospital there are physicians provide care for mothers unlike to the health centers in Ethiopian.

Even though using validated questionnaires and well trained data collectors could be mentioned as the strengths; the 24-h recall method may cause overestimation of the proportion of some IYCF practices due to recall and social desirability biases which could be reported as the limitation of this study.

## Conclusion

The majority of the mothers exclusively breastfed their child for the first six months and continue breastfeeding until two years. However appropriate complementary feeding practice was very low and there were mothers who gave prelacteal feeds and bottle fed their children. Educational status of the mother, attending postnatal care, place of delivery (hospital vs health center) and mass media exposure were independent predictors for complementary feeding practice. Hence, there is a need for strengthening the promotion of IYCF practice by health workers during postnatal care and using mass media giving emphasis for complementary feeding practice especially for mothers with lower educational status.

## Abbreviations

AOR: Adjusted Odds Ratio
CI: Confidence Interval
COR: Crude Odds Ratio
EBF: Exclusive Breast Feeding
EDHS: Ethiopian Demographic and Health Survey
IYCF: Infant and Young Child Feeding
SD: Standard Deviation
UNICEF: United Nations Children’s Fund
WHO: World Health Organization
